# Marriage Related Suicide Fatality Rates

**Published:** 2019-01

**Authors:** Fatemeh Akbarizadeh, Abdollah Hajivandi, Mohammad Hajivandi, Mohammad Sina Zeidabadinejad

**Affiliations:** 1 Mental Health Unit, Bushehr University of Medical Sciences, Bushehr, Iran.; 2 Department of Biostatistics and Epidemiology, Faculty of Health, Bushehr University of Medical Sciences, Bushehr, Iran.; 3 Department of Psychiatry, Faculty of Humanities Sciences, Bushehr Islamic Azad University, Bushehr, Iran.; 4 Nargund College of Pharmacy, Banglore, India.

**Keywords:** *Fatality Rates*, *Lethal*, *Marital Status*, *Psychiatry*, *Suicide*

## Abstract

**Objective:** The present study aimed at estimating the case fatality rates of suicide acts in different marital status subgroups and to find interrelation with a population living in Bushehr province along the coastal region of the Persian Gulf coast in southwest of Iran.

**Method** **:** In this analytical and observational study, suicidal data were gathered in 5 successive years (2008–2012) in the population of Bushehr. Data were extracted from suicide registration forms provided by the Psychiatric Health Unit of Ministry of Health. Questions mainly focused on demographic characteristics, including age, sex, literacy, residency, marital status, birth rank, the number of previous suicide attempts, and probable reasons of suicide, such as family conflicts or/and psychiatric complications. Fatality rates in different subgroups (male/female; married/unmarried; etc.) were compared and odds ratios were computed. The main limitation of this study was the lack of a specific grouping for those who cohabited together (Those who live together and have a sexual relationship without being married). The logistic regression model was used in data analysis.

**Results: **Case mortality rates of suicides were found to be 3.5% for both sexes, 5.2% in males and 2.7% in females. However, among the single population, either divorced or widowed, the probability of death due to suicide was 3.5 times higher (95% CI: 1.5–2.9) as compared to the unmarried. Age as a confounding factor in fatality rates made a significant difference between married and unmarried people, and differences disappeared after adjusting for age. However, fatality rate was still higher in the widowed /divorced group compared to other groups even after adjusting for age.

**Conclusion: **Fatality rate was higher in married people compared to the unmarried, however, after adjusting for age as a determinant factor, no significant difference was observed between the 2 groups. The highest death rate belonged to the age-specific widowed /divorced individuals as compared to all other marital status subgroups.

Suicide is one of the major causes of death in the world as more than 800 000 people die every year due to suicide (1). In 2015, suicide was introduced as the second leading cause of death among 15-29-year-olds globally (2). Approximately, 1 person dies every 40 seconds by committing suicide, which represents 1.8% of death cases in the world (3). Similar to many other disorders, suicide occurs based on sociodemographic characteristics, such as marital status. Emile Durkheim (4) conducted the first scientific study related to the interrelation of marital status and suicide, he also evaluated age specific suicide rates of married, widowed, and unmarried French people in both sexes evaluated age specific suicide rates of married, widowed, and unmarried French people in both sexes during 1889-1891. Suicide rates in single and married groups based on age and sex, suicide rates in widowed and married individuals, and the ratio of single to widowed cases were considered in that study. The suicidal rates of married persons were found to be much lower compared to single or widowed people in both sexes, particularly in males. Following his investigation, suicidal behaviors were placed as the topic of numerous studies in Western countries (4, 5) and in other non-Western nations (5, 6 and 7). 

A number of these studies focused on the possible confounding effects of other variables that might be related to the marital status and suicide incidence (8, 9). Findings of these studies confirmed that marriage is a protective factor against suicide (10), but there were some disagreements (11). 

According to the World Health Organization statistics, in 2013, the suicide mortality rate of Iranian people was estimated as 4.8 per 100 000 population (12). To date, several studies have investigated the key factors affecting suicide rates in Iran (6, 7). For instance, it was surveyed that completed and/or attempted suicides are more frequent among married persons than single ones in some parts of Iran (13). This might be in contrast to the popular belief that marriage has a protective role against psychological or behavioral disorders. The results of a meta-analysis indicated that family conflicts are a substantial factor associated with suicide in Iran (14). In Iran, traditional and religious beliefs almost urge young people to marry, but during the recent decade the ratio of marriage to divorce has changed drastically (15). 

In 2004, marriage to divorce ratio was recorded at 10, (one divorce in 10 marriages) while it decreased to 5.5 in the country in 2014, and in a city like Tehran, it was nearly 3.3, meaning that by each 33 marriages, 10 divorces were registered in this city (15). Nevertheless, family foundation and civil statues are yet important matters in Iranian culture. In 2014, a total of 774 513 marriages were registered all over the country, and 24% of couples aged under 25 (16). The results of Iranian census 2011 showed that 61% of women and 60% of men were married in Iran (15).

Bushehr province is a plain running area along the coastal region of the Persian Gulf in south-western Iran, with a population of about 1 500 000. In this province, suicide rates were reported as 5.8 and 2.9 per 100 000 in women and men, respectively, during 2010 (17). The annual suicidal rates in Bushehr were higher among women as compared to men at that time and also in nearly all previous and recent years. This study aimed to estimate the case fatality rates in suicide acts (Some suicide attempts led to death.) in different marital status subgroups and to portrait the probable connections.

## Materials and Methods

Data on suicide cases during 5 consecutive years (2008–2012) in Bushehr province were collected. A suicidal registry form was designed by the mental health bureau of Ministry of Health to collect information systematically about both non-fatal and fatal suicide acts. This form includes 2 sections, the first consists of questions on demographic characteristics of the persons who commit suicide (age, sex, literacy, residency, marital status, birth rank and the number of previous suicide attempts); and the second section was designed to determinate the probable reasons behind suicide attempts (family conflicts, psychiatric problems, addiction, poverty and prior suicidal behaviors of other family members). Such information forms are available in all hospitals as well as health and medical centers in the provinces. Input data are recorded by a trained registrar where the suicide take place. 

A consent declaration signed by suicidal person or his family members/relatives is required before completing the form. In addition, an official death certificate should reflect suicide as a cause of death, if suicidal attempt has led to death. In this regard, the International Classification of Diseases (Ninth Revision) has ruled to use specific codes named as E950–E959. An important inclusion criterion to be listed once a person commits suicide is age. In Iranian Ministry of Health, those attempted and/or commited suicides in persons under 10 years old are not considered as suicide case reports. Thus, we selected those who were 10 years and older. 

Hospitalized addicts that have been poisoned or overdosed by drugs, such as tramadol, are not postulated as suicidal cases. The completed suicide registry forms were transferred to the province health center, reviewed by responsible research groups, and data were archived in an Excel workbook for further analysis. Initially, the fatality rates of suicidal acts were computed for men and women separately in different marital status subgroups, and for those with different ages, the following categories were used: 10–25 years, 25–35 years, and 35 years and older. 

A logistic regression model was applied to the suicidal data to estimate the chance of death in the form of fatality rates in different marital status subgroups. Adjusted odds ratios (ORs) and 95% confidence intervals (CI) were calculated using unmarried individuals as the reference group. Odd ratios significantly higher than 1 indicated a higher fatality rate for the specific marital status as compared to the rates observed for unmarried people. The reference group was selected based on the lowest fatality rate that was perceived in unmarried people. From a tutorial point of view, unadjusted ORs were also computed to observe changes in magnitude after adjusting for other variables in this study model, such as age and sex. 

## Results

During 2008–2012, a total of 141 successful and 3889 attempted suicides were registered in Bushehr province health administration, an average of 28 per year for fatal completed suicides and 778 per year for non-fatal attempted suicides. The suicidal case fatality rate for 5 years was 3.5% for both sex, 5.2% for men, and 2.7% for women. Case fatality rate in men was about 2-fold higher than women, while the number of suicides in women reached to 72 cases, a little greater than 69 suicides in men. The number of failed suicide attempts in women was also higher than that of men (2618 women vs 1265 men). The minimum mortality rate of these 5 years was calculated as 2.2 in 2008, and the maximum was 6.3 in 2009 in both sexes. 

Table 1 demonstrates the number of completed and failed suicide attempts and case fatality rates by sex and marital status in different age groups. In an ascending manner, widowed/divorced women were evidenced to have the highest suicidal fatality rate, followed by married men above 35 years old, compared to other experimental groups in both sexes. Married individuals possessed higher fatality rates compared to unmarried people in both sexes. The last age group was 35- year- old group as 35 was the 92th percentile of all ages, meaning that only 8% of the population under study aged 35 years and older.

In all marital status groups, the highest fatality rates were recorded in the last age group (35 years old and older) in both sexes, except for that of divorced men, for whom no suicide and/or only a few cases of unsuccessful attempts were registered. The highest fatality rate of 42% was observed in divorced/widowed women group aged 35 years and older.

Table 2 shows the results of the logistic regression model fitted to the variables that were statistically significant or at least showed a trend towards significance in the bivariate analysis with results of suicide acts, which either led to death or not. The results of bivariate analysis were also shown in the form of unadjusted ORs, when there was only 1 independent variable in the logistic regression model. Adjusted ORs for each variable was computed by omission of confounding effects of 2 other independent variables incorporated in the model.

Overall, age was the strongest risk factor affecting death incidence in suicidal behaviors among other continuous variables, whereas marital status had a more effect in accompany of categorical variables. The probability of death among those aged 35 and older was 5 times higher than those aged 25 and younger (95%, CI: 3.4.–8.3). This probability was 3.5 times higher (95%, CI: 1.5.–2.9) in the divorced or widowed compared to the unmarried. Bivariate analysis estimated that death rate in married persons was 1.7 times higher than unmarried, but following adjustment forage- specific confounding effects, the relation disappeared (OR = 0.96; 95%, CI: 0.65 – 1.40), which means higher fatality rate was primarily associated with age rather than marital status. In other words, fatality rates of suicide acts in married individuals were higher than that of unmarried due to the higher mean of age factor in the married group.

**Table 1:** Number of Completed and Attempted Suicide and Case Fatality Rate by Sex and Marital Status in Different Age Groups

**Sex**	**Marital Status**	**Age**	**Suicide**	**Attempted Suicide**	**Case Fatality Rate (%)**
Men	Unmarried	10 – 24	30	761	3.8
		25 – 34	6	142	4.1
		35+	4	12	25.0
	Married	10 – 24	5	125	3.8
		25 – 34	13	140	8.5
		35+	20	84	19.2
	Widowed/ Divorced	10-24	0	1	0
		25-34	0	1	0
		35+	0	1	0
Women	Unmarried	10 – 24	28	1159	2.4
		25 – 34	2	95	6.9
		35+	3	9	25.0
	Married	10 – 24	18	714	2.5
		25 – 34	18	469	3.7
		35+	17	188	8.3
	Widowed/ Divorced	10-24	2	17	10.5
		25-34	1	18	5.3
		35+	6	8	42.9

**Table 2:** Not Adjusted and Adjusted Odds Ratio and 95% Confidence Interval of Fatality Rates of Suicide Acts for Different Variables

**Variables**	**Not Adjusted OR (95% CI**	**P value**	**Adjusted OR (95% CI)**	**P- value**
Marital status				
Unmarried	1		1	
Married	1.78 (1.29 – 2.44)	0.000	0.96 (0.65 – 1.40)	0.818
Widowed/divorced	7.25 (3.37 – 15.6)	0.000	3.47 (1.52 – 7.93)	0.003
Age				
(10 – 24)	1	0.003	1	
(25 – 34)	1.76 (1.22 – 2.53)	0.000	1.72 (1.14 – 2.57)	0.009
35^+^	5.56 (3.86 – 8.01)		5.35 (3.43 – 8.34)	0.000
Gender				
Female	1		1	
Male	1.67 (1.24 – 2.27)	0.001	1.70 (1.24 – 2.36)	0.001

## Discussion

Results of this study revealed that the fatality rates of suicide acts differ among marital status groups in the study population. The lowest fatality rate in both sexes was detected among unmarried women, while the highest rate was among divorced or widowed women. In both sexes, mortality rates were higher among married persons than the unmarried persons, especially the difference was more than 2-fold in married men compared to unmarried men. Overall, the highest fatality rate was among divorced women, followed by widowed women and unmarried women above 35 years old. According to the findings of the present study, 1 in 5 suicide attempts in divorced women led to death. Several studies showed that suicidal fatality rates increases with age. We also found the same relation in our data (18, 19). In each marital status group of the two sexes, we computed the mortality rate of suicide among three different age groups. Fatality rates increased with age; thus, it might be concluded that age might be a potentially confounding factor for correlations between marital status and the fatality rate of this study. In other words, fatality rate in married people is higher than the unmarried by reason of married groups on average are older than the unmarried, and not as a result of their marital status. 

The logistic regression model fitted to the data confirmed the hypothesis that there is no significant difference between the mortality rates of suicide in married and never married persons. Most previous studies suggested that marriage is a protective factor for suicide act in two/both sexes while it is more impressive in males. An investigation performed in Denmark indicated that suicidal rates among singles were two-fold higher than married people in both sexes (19). Also, it was stated that being married seems to be a protective factor against suicide, but the impact of being never married, divorced/separated or widowed varies with age and gender (20). A European comparative study exhibited that suicide risk in non-married people was at least 2-fold higher than in married people in many European countries (21).The results of a population-based prospective cohort study on Japanese population aged 40–64 years demonstrated that marital status was significantly associated with the risk of committing suicide only in men, suicide mortality rates of males were 2.5 times higher than females (22). In contrast, some other studies suggest that suicidal acts occur more in married persons than in the unmarried ones. The majority of these studies belong to non-Western countries. 

In Ilam province in Iran, a higher suicide rate, fatal, or incomplete attempts was registered among married persons than those who were never married (13). Results of a study conducted in Mumbai- India proved that marriage and family conflicts are related to suicidal behaviors (23).The world report on violence and health in 2002 concluded that marriage is generally protective against suicide in industrialized countries, but it is not a strong barrier in contradiction of suicide attempts in developing countries (24). Lethality of suicidal acts and other related factors have also been investigated although the majority focused on age and gender. The likelihood of death in men who committed suicide was reported higher than women in nearly all case studies.

An American study showed that 23% of suicide acts proved lethal among men, which was four times higher than mortality rates in women (24). In 1 study, it was revealed that American women were more likely to commit suicide than men, but the suicidal attempts of men were almost five times more likely to be fatal than this act among women (18). However, in our study, men’s fatality rates of suicide acts were about 2 times greater than that of women, once adjusted for age and marital status. Radhakrishnan, in his article, indicated that quality of marital relationship, emotional warmth, extended family support, and ability to handle marriage-related and child-rearing stress are more important than marital status (10) even though they are difficult to study. The main limitation of our study was the lack of a specific grouping for those cohabiting together.

## Limitation

The main limitation of our study was the lack of a specific grouping for those cohabiting together. Those who live together and have a sexual relationship, and their marriage is not officially registered.

## Conclusion

Suicide fatality rate was higher in married people compared to the unmarried, however, after adjusting for age as a determinant factor, no significant difference was observed between the 2 groups. The highest death rate belonged to the age-specific widowed /divorced women as compared to all other marital status subgroups, majority of these women live alone that increases risk of death after suicide. The lowest death rate belonged to unmarried young men, but the number of this group in our study was very small, then the estimated rate could not be reliable. 
